# Nano-Encapsulated Cumin Oil and *Bacillus subtilis* Enhance Growth Performance, Immunity, Oxidative Stability, and Intestinal Integrity in Growing Rabbits Under High Ambient Temperature

**DOI:** 10.3390/vetsci12111039

**Published:** 2025-10-28

**Authors:** Ahmed M. Elbaz, Hind Althagafi, Ahmed Samy, Ahmed Sabry Arafa, AbdelRahman Y. Abdelhady, Ahmed M. Elkanawaty, Khairiah Mubarak Alwutayd, Saad Shousha, Abdelrahman M. Hereba, Ahmed Ibrahim El Sheikh, Salah Abdulaziz AL-Shami, Sherief M. Abdel-Raheem, Mahmoud HA Mohamed, Mohammed Al-Rasheed, Ahmed Ateya, Mohamed Marzok

**Affiliations:** 1 Animal and Poultry Nutrition Department, Desert Research Center, Mataria, Cairo 11753, Egypt; 2 Department of Biology, College of Science, Princess Nourah bint Abdulrahman University, P.O. Box 84428, Riyadh 11671, Saudi Arabia; hjalthagafi@pnu.edu.sa (H.A.);; 3 Department of Animal Production, National Research Centre, Giza 12622, Egypt; asamy1@yahoo.com; 4 Poultry Nutrition Department, Animal Production Research Institute, Agricultural Research Center, Ministry of Agriculture, Giza 12622, Egypt; ahmedsabry.as@gmail.com; 5 Poultry Production Department, Faculty of Agriculture, Ain Shams University, Cairo 11759, Egypt; 6 Poultry Breeding Department, Animal Production Research Institute, Agricultural Research Center, Ministry of Agriculture, Giza 12622, Egypt; 7 Department of Biomedical Sciences, College of Veterinary Medicine, King Faisal University, Al-Hofuf 31982, Saudi Arabia; smmshousha@kfu.edu.sa; 8 Department of Microbiology, College of Veterinary Medicine, King Faisal University, Al-Hofuf 31982, Saudi Arabia; ahereba@kfu.edu.sa; 9 Department of Public Health, College of Veterinary Medicine, King Faisal University, P.O. Box 400, Al-Hofuf 31982, Saudi Arabia; aelsheikh@kfu.edu.sa (A.I.E.S.); salshami@kfu.edu.sa (S.A.A.-S.); sdiab@kfu.edu.sa (S.M.A.-R.); 10 Department of Clinical Sciences, College of Veterinary Medicine, King Faisal University, P.O. Box 400, Al-Hofuf 31982, Saudi Arabia; mhmohammad@kfu.edu.sa (M.H.M.); malrasheed@kfu.edu.sa (M.A.-R.); 11 Department of Development of Animal Wealth, Faculty of Veterinary Medicine, Mansoura University, Mansoura 35511, Egypt

**Keywords:** heat stress, rabbits’ performance, probiotics, essential oils, antioxidant status, gut microbiota, gene expression

## Abstract

**Simple Summary:**

Heat stress causes significant economic loss due to its multiple adverse effects on the health and performance of rabbits, including gastrointestinal disturbances, impaired immunity, oxidative stress, and organ damage, leading to deteriorating production performance. Nutritional strategies, such as dietary supplements, have proven their effectiveness in mitigating the negative effects of heat stress on animals. Therefore, the primary objective of this study was to evaluate the synergetic effect of adding *Bacillus subtilis* (*B. subtilis*) and nano-encapsulated cumin oil to mitigate the impacts of heat stress on the performance and health of growing rabbits. Incorporating *B. subtilis* and nano-encapsulated cumin oil into rabbit diets may mitigate the negative effects of heat stress by improving growth performance and feed efficiency, increasing antioxidant capacity and digestive enzyme activity, and modifying gut microbiota and gene expression associated with gut health. Therefore, it represents a promising nutritional strategy for rabbits under high temperatures.

**Abstract:**

The study evaluated the influence of dietary supplementation with nano-encapsulated cumin oil, *B. subtilis*, or a combination of both to mitigate the impacts of heat stress on the performance and health of growing rabbits. In the feeding trial, a total of eighty-four growing New Zealand White (35 days, 781.3 ± 1.8 g average body weight) were randomly distributed in a completely randomized design into four groups; each had 21 rabbits arranged in 7 replicates (3 rabbits each). The experiment lasted 42 days (35 days to 77 days). Growing rabbits received a basal diet (first group, CON) without additives, while the other groups were supplemented with nano-encapsulated cumin oil (NECO, 200 mg/kg), *B. subtilis* (BS, 500 mg/kg), or both (BSNO, 500 mg BS plus 200 mg/kg NECO). Adding BSNO significantly enhanced body weight gain, carcass weight, and feed conversion ratio and reduced mortality rate (*p* < 0.05). Additionally, the BSNO enhanced digestive system performance by increasing the secretion of trypsin enzymes, as well as nutrient digestibility, especially for protein and fiber (*p* < 0.05). Supplementing BSNO enhanced oxidative stability and immunity via higher levels of superoxide dismutase (SOD), IgA, IgG, triiodothyronine (T3), thyroxine (T4) and lower malondialdehyde (MDA) levels (*p* < 0.05), indicating a better ability to adapt to stress. During the examination of gut health, pathogenic bacteria counts decreased, as well as down-regulation of interleukin-6 (IL-6) gene expression and up-regulation of cationic amino acid transporter-1 (CAT-1), interleukin-10 (IL-10), and mucin-2 (MUC-2) gene expression (*p* < 0.05), supporting gut integrity. This study highlights the potential of mixing nano-encapsulated cumin oil and *B. subtilis* in growing rabbits’ diets as an effective strategy to counteract the negative effects of heat stress caused by high ambient temperatures.

## 1. Introduction

Environmental stress is one of the constraints to animal production [1]. Among these pressures is heat stress, which causes numerous adverse health effects on rabbits, resulting in significant economic losses in the rabbit industry [2]. Rabbits are unable to dissipate excess heat due to their limited number of sweat glands, making them more susceptible to heat stress. Oxidative stress, impaired immunity, and endocrine deregulations [1], as well as reproductive disorders and organ damage [3], are among the most important symptoms of heat stress in rabbits, which decline in their production performance and an increase in the mortality rate [4]. Additionally, rabbit breeders suffer from some problems during the weaning period, including a high mortality rate, which is attributed to intestinal disorders and impaired immunity [5]. The transition of rabbits from liquid milk to solid feed during the weaning period can be one of the main causes of intestinal disorders such as flatulence, diarrhea, or constipation [6]. Recently, feeding strategies have been a major factor in mitigating heat stress and weaning problems [2,3,7]. Nutritional manipulation through the use of functionally effective additives has played a positive role in resolving many of the obstacles facing rabbit breeding, such as herbs, the addition of vitamins, minerals, essential oils, probiotics, organic acids, etc. [8,9,10,11].

Probiotics are among the feed additives that have become widely used in animal feed due to their many benefits [8,9]. *B. subtilis* is a key component of most probiotic formulas (withstands harsh conditions) due to its many properties, including antimicrobial, anti-inflammatory, blood metabolic profile, and immunomodulatory properties [12,13], as well as the production of some enzymes that enhance feed efficiency [14]. Additionally, it modulates the metabolism of amino acids and vitamin B [13] and enhances disease resistance [12,15].

Several recent reports have revealed the benefits of incorporating medicinal and aromatic plants and their products (essential oils) as sustainable and natural alternatives to traditional feed additives in rabbit nutrition [14]. Analyses of various medicinal and aromatic plants have revealed a diverse array of biologically active compounds that play a key role in the numerous positive properties [8,16] that have the potential to enhance rabbit health. The proven properties of these biologically active compounds include antioxidant, antimicrobial, and anti-inflammatory properties [14]. *Cuminum cyminum* L. is a distinctively flavored herb belonging to the *Apiaceae* family [17]. *Cuminum cyminum* L. and its essential oil contain Cuminaldehyde and p-Cymene, γ-Terpinen, the biologically active compound responsible for most of the oil’s properties [18], which include antibacterial, antioxidant, and antifungal properties [17].

In recent years, there has been an increased focus on nanotechnology due to its many benefits, including enhanced bioavailability, stability, cellular uptake of nutrients, and solubility [7,19], which make it more effective than the base material. This is similar to the case with essential oils, where numerous studies have documented the positive role of converting pure essential oils to nano-emulsified essential oils [17], making them more effective as animal feed additives [18,19] by enhancing the antioxidant and antibacterial properties of essential oils.

Nutritional manipulation through a feed additives strategy had a positive effect on mitigating environmental stress and disease resistance in animals. Therefore, this study aimed to use *B. subtilis* and nano-emulsified cumin essential oil to alleviate the effects of heat stress and weaning problems in weaned rabbits. This combination was selected to evaluate the potential synergistic effects in alleviating the impacts of heat stress in rabbits, as *B. subtilis* may enhance intestinal colonization and enzyme secretion [12,13], while essential oils provide antimicrobial and antioxidant protection [19], together supporting better intestinal integrity and metabolic function, as well as enhancing performance in rabbits under heat stress. Thence, the current study evaluated the effect of supplementing *B. subtilis* and nano-emulsified cumin oil and their blend on growth performance, carcass characteristics, hematological parameters, immune response, gut microbiota, and gene expression.

## 2. Materials and Methods

### 2.1. Preparation of Materials

*Bacillus subtilis* was obtained from the Microbiology Department, Faculty of Agriculture, Ain Shams University, Egypt. Nano-encapsulated cumin essential oil was synthesized by the author from the National Research Centre, Egypt. From pure life for investment and agricultural development in Cairo, Egypt, cumin essential oil (CEO) was purchased. Tween 80 and sodium tripolyphosphate (TPP) were purchased from Sigma Chemicals, St. Louis, MO, USA.

### 2.2. Synthesis of Nano-Encapsulated Cumin Oil (NECO)

The nano-encapsulation of cumin essential oil (CEO) was carried out using the ionic gelation method according to López-Meneses et al. [20]. Chitosan (1 mg/mL; derived from crab shells) was dissolved in a water bath at 60 °C in 1% (*v*/*v*) acetic acid solution with constant stirring until complete dissolution to obtain a 1.0 mg/mL stock solution, and the pH was adjusted to 4.8 using a solution of NaOH (3 N), then Tween 80 was added into the chitosan solution under magnetic stirring (800 rpm) for 10 min. The CEO was then gradually incorporated into the mixture with continuous stirring for half an hour. Thereafter, dropwise (1 mL/min) addition of sodium tripolyphosphate (TPP) solution (1.0 mg/mL in deionized water) was added at a 4:1 (*w*/*w*) chitosan: TPP ratio while being constantly stirred for 30 min/RT. To improve homogeneity and decrease particle size, the resultant suspension was sonicated for 10 min using an ultrasonic probe sonicator. To guarantee the nanoparticles’ suitability for dietary use in rabbits, every step of the preparation process was carried out in a sterile environment. The chemical composition of cumin essential oil (CEO) was investigated using gas chromatography–mass spectrometry (GC–MS), and specific constituents were identified by contrasting their mass spectra with reference spectra as per Bai et al. [21] (Table 1). A Nano-ZS ZEN analyzer (Malvern Instruments, Malvern, UK) was used to characterize the generated nanoemulsion to measure the particle size, zeta potential, and polydispersity index (PDI). Having a zeta potential of +24.2 mV, a PDI of 0.183, and an average droplet size of 92.3 nm, the produced nanoparticles demonstrated satisfactory stability and uniform distribution at the nanoscale.

### 2.3. Rabbits, Experimental Design, and Diet

The experiment animals were provided from Ras Sadr Station, Desert Research Center, and the experiment was conducted in accordance with the Animal Care and Experimental Research Ethics Committee of the Faculty of Agriculture, Ain Shams University, and Desert Research Center, Egypt. All protocols were implemented in accordance with the International Guideline for the Protection of Animals Used for Scientific Purposes.

In a feeding experiment that started at 35 days of old (average body weight 781.3 g) and continued until 77 days of old, eighty-four growing New Zealand White (NZW) rabbits were divided into four groups (21 rabbits/group), and each of seven replicates (3 rabbits/replicate) was randomly assigned. The first group (control, CON) was fed on a basal diet, the second, third, and fourth groups were fed on the basal diet added with *B. subtilis* (BS, 500 mg/kg), nano-encapsulated cumin oil (NECO, 200 mg/kg), and a mixture of *B. subtilis* and nano-encapsulated cumin oil (BSNO, 500 mg and 200 mg/kg diet, respectively). The selected doses were based on previous studies showing beneficial effects of similar levels of *B. subtilis* [22] and essential oils [23] on growth performance, gut health, and antioxidant status in poultry. A pelleted diet and clean, fresh water were offered ad libitum. Growing rabbits were reared during the summer at Ras Sadr Station, Desert Research Center, Egypt. The nutritional needs of the experimental rabbits were met by formulating experimental diets according to the recommendations of the National Research Council [24] (Table 2). The required amount of nano-encapsulated cumin oil and *B. subtilis* (1 × 10^6^ CFU/g feed) spores was blended with a small portion of the basal feed to form a premix. This premix was then thoroughly mixed with the remaining feed using a mechanical mixer to ensure uniform distribution, before pelleting. The treated feed was prepared weekly to maintain the stability and activity of the additives and stored in airtight containers at room temperature under dry conditions. To calculate the temperature-humidity index (THI) [25], daily temperature and humidity were recorded at 10:00 a.m. and 4:00 p.m. The THI was used to determine the rabbits’ stress levels: <27.8 indicates no heat stress, 27.8 to 28.9 indicates moderate heat stress, 29.0 to 30.0 indicates severe heat stress, and >30.0 indicates extremely severe heat stress. According to Shebl et al. [26] one rabbit or duplicate’s respiratory rate (RR/min) and pulse rate (PR/min) were determined by counting the movements of the chest fleece and using a finger to count the pulses in the femoral artery, respectively.

### 2.4. Performance Index

At 56 and 77 days of age, live body weight (LBW), Daily feed intake (DFI), and daily mortality were noted. Body weight gain (BWG) and feed conversion ratio (FCR) were computed. Mortality was monitored daily throughout the 6-week experimental period. Dead rabbits were immediately removed, and any signs of disease or injury were documented. The number of surviving rabbits in each pen was recorded weekly to calculate cumulative mortality and survival rates. In measuring performance and mortality rate, the pen was considered the experimental unit. One rabbit was chosen at random from each replicate in the experimental group (seven rabbits per group) at the end of the 6-week experiment, weighed separately, and slaughtered for carcass examination. At the end of the experiment (77 days), one rabbit was randomly selected from each replicate in the experimental group (five rabbits/group), weighed individually, and slaughtered to evaluate the targeted experimental parameters. The carcass, lungs, liver, kidneys, heart, and giblets were weighed, and the total edible parts were calculated based on Ghosh and Mandal [27]. Additionally, after the experiment concluded, one rabbit/replicate (seven from the group) was placed in metabolic cages. Before the collection period, the rabbits were kept for 24 h to acclimatize. After four days of collection, the feces were ground up, dried at 65 °C for 48 h, and kept in polyethylene bags at −10 °C until they could be subjected to chemical tests. In accordance with AOAC procedures, the feed and feces were examined for dry matter, crude fiber, ether extract, crude protein, and nitrogen-free extract [28]. Samples were obtained from the duodenum at 77 days of age at slaughter in order to measure the activity of digestive enzymes. According to Abdel Moneim et al. [29], specific commercial kits (Nanjing Jiancheng Bioengineering Institute, Nanjing, China) were used to measure the activities of trypsin, cellulase, and amylase.

### 2.5. Biochemical Analysis

Blood samples were taken in anticoagulant-free tubes during slaughtering (at 77 days of age), centrifuged for 15 min at 3000× *g*, and serum was taken and kept at −20 °C. High-density lipoprotein (HDL), low-density lipoprotein (LDL), cholesterol, glucose, triglycerides, total protein, and albumin levels were determined calorimetrically using an auto-analyzer system by using commercially available kits (Spinreact Co., Ltd., Girona, Spain). Alanine aminotransferase (ALT) and aspartate aminotransferase (AST) levels were measured to assess liver function. Thyroxine (T4) and triiodothyronine (T3) were assayed using radioimmunoassay (RIA) kits as described by Ibrahim et al. [30]. The commercial kits from Bio Diagnostic Company (Giza, Egypt) were used to specify the levels of glutathione peroxidase (GPx), malondialdehyde (MDA), and superoxide dismutase (SOD) in the serum. In addition, using ELISA kits, blood concentrations of IgG, IgM, and IgA were estimated (Life Diagnostics Inc., West Chester, PA, USA).

### 2.6. Cecum Microbial Enumeration

At slaughter, 10 g of the cecum of seven experimental rabbits/group was collected, and the samples were stored at −20 °C after being placed in sterile bags until the required analysis. Necessary dilutions were made of the cecal samples and grown on agar suitable for each microbe under the required temperature and aerobic or anaerobic conditions. *Lactobacillus*, *Escherichia coli* (*E. coli*), and *Clostridium perfringens* (*C. perfringens*) bacteria were enumerated (MRS agar, Egg yolk emulsion (50%), and MacConkey agar, respectively). The microbiota count was calculated as log 10 colony-forming units per gram of cecal digesta.

### 2.7. mRNA Gene Expression

At the end of the experimental period, 28 growing rabbits (7 rabbits per group, injected with sodium pentobarbital) were sacrificed for cecal tissue sampling to estimate the effect of experimental supplementation on the gene expression of both cationic amino acid transporter-1 (CAT-1) and mucin-2 (MUC-2), in addition to cytokines including interleukin-6 (IL-6) and interleukin-10 (IL-10). Total RNA suspension was extracted from the cecal membranes and then homogenized, visualized, and quantified using a Nanodrop spectrophotometer (BMG Lab Tec. GmbH, Offenburg, Germany); all procedures were performed according to the manufacturer’s instructions. After the amplification of cDNA, the specificity of the amplification was assessed by performing a dissociation curve of the real-time polymerase chain reaction (7500 Fast Real-time PCR) products. The forward and reverse primers for CAT-1 were F: CCAGTCTATTAGGTTCCATGTTCC and R: CGATTATTGGCGTTTTGGTC (Accession number XM_002721425.3); MUC-2, F: TATACCGCAAGCAGCCAGGT and R: GCAAGCAGGACACAGACCAG (Accession number L41544.1); IL-10, F: AAAAGCTAAAAGCCCCAGGA and R: CGGGAGCTGAGGTATCAGAG (NM_001082045.1) for IL-6, F: ACGATCCACTTCATCCTGCG and R: GGATGGTGTGTTCTGACCGT (NM_001082064.2). Additionally, GAPDH, F: TGTTTGTGATGGGCGTGAA and R: CCTCCACAATGCCGAAGT (DQ403051.1). The 2^−ΔΔCT^ method was used to determine the relative expression levels of MUC-2, CAT-1, IL-6, and IL-10 genes [31].

### 2.8. Statistical Analysis

The data were analyzed by ANOVA using the general linear model procedure of SPSS (19.0) as randomized complete design (CRD). Following a significant F-test, Tukey’s post hoc test was employed to determine the statistically significant differences between the experimental groups under heat stress. Survival data were analyzed using Kaplan–Meier estimates and compared among treatments with a log-rank test, while pen-level mortality proportions were evaluated using the Kruskal–Wallis test, considering the pen as the experimental unit. Shapiro–Wilk tests were used to assess homogeneity and normality between experimental groups, respectively. Since *p* < 0.05, these differences were considered significant.

## 3. Results

### 3.1. Rabbit Welfare

**Figure 1:**
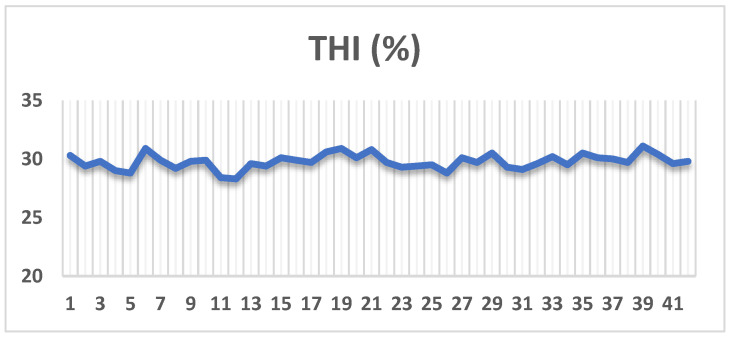
Temperature-humidity index (THI).

The rabbits were exposed to heat stress, as indicated by the THI value, which varied between 29.2 and 30.3 during the entire experimental period (Figure 1). The HR and RR analysis was performed on 24 male rabbits (7 rabbits/group), with the range for HR was 152 to 197 beats/min, while the range for RR was 81 to 106 breaths/min.

### 3.2. Performance

**Figure 2:**
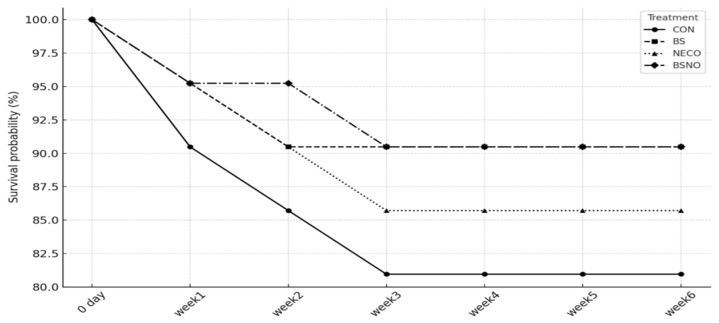
Effect of *B. subtilis*, nano-encapsulated cumin oil, and their mixture supplementation on mortality percentages of growing rabbits. CON: rabbits fed a basal diet without a feed additive (control diet), BS: control diet plus *B. subtilis* (500 mg/kg), NECO: control diet plus nano-encapsulated cumin oil (200 mg/kg), BSNO: control diet plus *B. subtilis* and nano-encapsulated cumin oil. Kaplan–Meier survival over six weeks for CON, BS, NECO, and BSNO. No overall difference among curves (log-rank χ^2^ = 1.52, df = 3, *p* = 0.6765).

Results of the performance index (growth and carcass traits) of rabbits fed *B. subtilis*, nano-encapsulated cumin oil, and their mixture during heat stress are shown in Table 3 and Table 4. During all experimental phases, BWG decreased and FCR deteriorated (*p* < 0.05), while daily feed intake was unaffected in rabbits that did not receive experimental supplements under heat stress (Table 3). During the periods from 35 to 56 days, DFI was not affected, while BWG tended to increase in the BS, NECO, and BSNO groups (*p* < 0.05) compared to the control group. However, FCR significantly decreased in the BSNO group compared to the other groups. During the periods of 57–77 days, BWG increased in the BS, NECO, and BSNO groups (*p* < 0.05) compared to the control groups, while the highest BWG was in the BSNO group. In the same context, FCR significantly decreased in rabbits receiving BS and BSNO compared to the NECO and control groups (*p* < 0.05), while DFI was not affected. During the total experimental period (35–77 d), BWG significantly increased in the BS and BSNO groups (*p* < 0.05) compared to the control and NECO groups, while DFI remained unchanged between the experimental groups (*p* < 0.05). In addition, FCR was significantly decreased in rabbits receiving BS, NECO, and BSNO compared to the control group; however, the best FCR was in rabbits receiving BSNO. Additionally, carcass weight increased in rabbits receiving BSNO compared to the other groups (*p* < 0.05); however, other carcass characteristics, such as heart, kidney, lung, liver, giblets, and TEP, were not affected by the experimental treatments (Table 4). Furthermore, the mortality rate was significantly (*p* < 0.05) reduced in rabbits receiving BS and BSNO during the entire rearing period compared to the control group (Figure 2). Cumulative mortality over six weeks ranged from 9.5% to 19.0% (CON 19.0%; NECO 14.3%; BS 9.5%; BSNO 9.5%; *n* = 21/treatment). Kaplan–Meier survival at week 6 was 81.0% (CON), 85.7% (NECO), and 90.5% (BS and BSNO). Survival curves did not differ significantly among treatments (log-rank χ^2^ = 1.52, df = 3, *p* = 0.6765). Pen-level mortality proportion also showed no treatment effect (Kruskal–Wallis H = 3.40, df = 3, *p* = 0.3340). Directionally, CON had the highest losses, whereas BS and BSNO showed the lowest losses, with NECO intermediate.

### 3.3. Digestive System Performance

**Figure 3:**
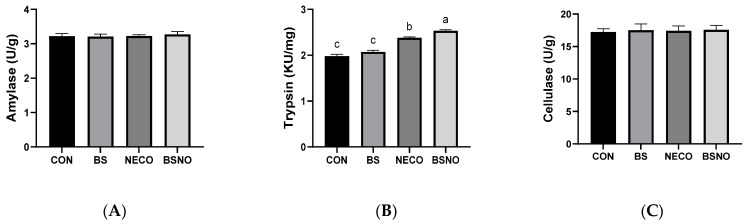
Effect of *B. subtilis*, nano-encapsulated cumin oil, and their mixture supplementation on digestive enzyme activity (amylase (**A**), trypsin (**B**), and cellulase (**C**)) of growing rabbits at 77 d. CON: a basal diet without a feed additive (control diet), BS: control diet plus *B. subtilis* (500 mg/kg), NECO: control diet plus nano-encapsulated cumin oil (200 mg/kg), BSNO: control diet plus *B. subtilis* and nano-encapsulated cumin oil. Values with different superscript letters are statistically different.

Results of the digestive system performance (digestibility coefficients and digestive enzyme activity) of rabbits fed *B. subtilis*, nano-encapsulated cumin oil, and their mixture during heat stress are shown in Table 5 and Figure 3A–C. Dry matter digestibility increased in rabbits receiving BS and BSNO compared to rabbits receiving CON and NECO groups (*p* < 0.05). Additionally, crude protein and crude fiber digestibility increased (*p* < 0.05) in rabbits receiving BSNO, BS, and NECO compared to the control group; whereas, the best digestibility of crude protein and crude fiber was in rabbits receiving BS and BSNO (*p* < 0.05). However, the digestion of ether extract and NFE was not affected (*p* < 0.05) among rabbits in the experimental groups. The activity of digestive enzymes, such as cellulase and amylase, was not affected by the experimental treatments (*p* < 0.05), except for trypsin, which significantly increased in rabbits receiving NECO and BSNO compared to those receiving CON and BS.

### 3.4. Serum Biochemistry

**Figure 4:**
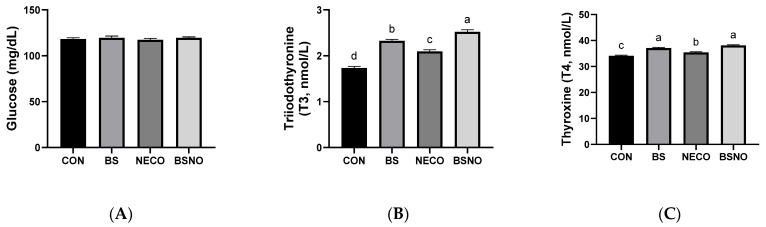
Effect of *B. subtilis*, nano-encapsulated cumin oil, and their mixture supplementation on glucose and thyroid activity (glucose (**A**), triiodothyronine (T3, (**B**)), and thyroxine (T4, (**C**)) of growing rabbits at 77 d. CON: a basal diet without a feed additive (control diet), BS: control diet plus *B. subtilis* (500 mg/kg), NECO: control diet plus nano-encapsulated cumin oil (200 mg/kg), BSNO: control diet plus *B. subtilis* and nano-encapsulated cumin oil. Values with different superscript letters are statistically different.

Results of the biochemical analysis (lipid profile, liver integrity, and stress index) of rabbits fed *B. subtilis*, nano-encapsulated cumin oil, and their mixture during heat stress are shown in Table 6 and Figure 4A–C. Total protein levels increased in rabbits fed NECO and BSNO compared to the other groups (*p* < 0.05). Likewise, HDL levels also increased in rabbits fed BS, NECO, and BSNO compared to the control group (*p* < 0.05), while triglycerides and cholesterol levels decreased in rabbits fed BS, NECO, and BSNO compared to the control group (*p* < 0.05, Table 6). Nevertheless, albumin and LDL levels were unaffected between experimental treatments. To assess liver health, ALT and AST levels were measured; our results showed a decrease in AST levels in rabbits receiving BSNO, NECO, and BS compared to the control groups; however, the lowest AST level was in the BSNO group. However, ALT levels were not affected among experimental treatments (*p* < 0.05, Table 6). Among the stress markers that showed improvement with the addition of experimental supplements were thyroxine (T4) and triiodothyronine (T3). The results showed an increase in T4 and T3 levels in rabbits fed BS, NECO, and BSNO compared to the control group, while glucose levels were unaffected between the experimental groups (*p* < 0.05, Figure 4).

### 3.5. Immuno-Antioxidant Status

**Figure 5:**
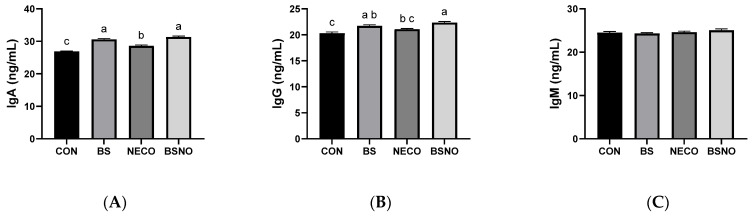
Effect of *B. subtilis*, nano-encapsulated cumin oil, and their mixture supplementation on immune status (IgA (**A**), IgG (**B**), and IgM (**C**)) of growing rabbits at 77 d. CON: a basal diet without a feed additive (control), BS: control diet plus *B. subtilis* (500 mg/kg), NECO: control diet plus nano-encapsulated cumin oil (200 mg/kg), BSNO: control diet plus *B. subtilis* and nano-encapsulated cumin oil. Values with different superscript letters are statistically different.

**Figure 6:**
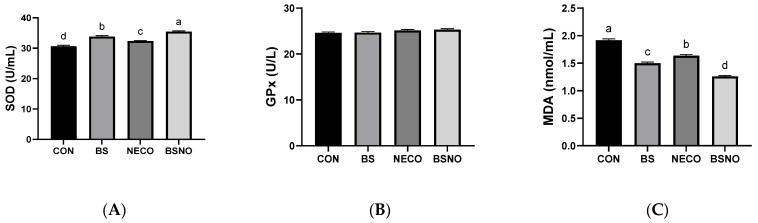
Effect of *B. subtilis*, nano-encapsulated cumin oil, and their mixture supplementation on oxidative stress indicators (superoxide dismutase (SOD, (**A**)), glutathione peroxidase (GPx, (**B**)), and malondialdehyde (MDA, (**C**)) of growing rabbits at 77 d. CON: a basal diet without a feed additive (control diet), BS: control diet plus *B. subtilis* (500 mg/kg), NECO: control diet plus nano-encapsulated cumin oil (200 mg/kg), BSNO: control diet plus *B. subtilis* and nano-encapsulated cumin oil. Values with different superscript letters are statistically different.

Figure 5 and Figure 6 show the effect of *B. subtilis*, nano-encapsulated cumin oil, and their mixture on immune status and oxidative stress indicators. IgA levels increased in the BS, NECO, and BSNO groups compared to the control group (*p* < 0.05, Figure 5A). Similarly, IgG levels increased in the BS and BSNO groups compared to the control and NECO groups (*p* < 0.05); however, IgM levels were not affected among the experimental groups (Figure 5B,C). Additionally, SOD activity significantly increased and MAD levels decreased in rabbits fed a diet containing BS, NECO, and BSNO compared to rabbits fed a control diet (*p* < 0.05), while GPx levels were unaffected by the experimental treatments (Figure 6A,B). Rabbits receiving BSNO also showed higher SOD activity and lower MAD levels than the other groups (*p* < 0.05, Figure 6C).

### 3.6. Microbial Enumeration

Table 7 shows the effect of experimental supplements on the intestinal microbial content of rabbits exposed to heat stress. *Lactobacillus* counts increased and *E. coli* counts decreased in the BS, NECO, and BSNO groups compared to the control group (*p* < 0.05), while the lowest *E. coli* counts and highest *Lactobacillus* content were in the BSNO group (*p* < 0.05). Similarly, *C. perfringens* counts decreased (*p* < 0.05) in the BS and BSNO groups compared to the control and NECO groups.

### 3.7. Gene Expression

**Figure 7:**
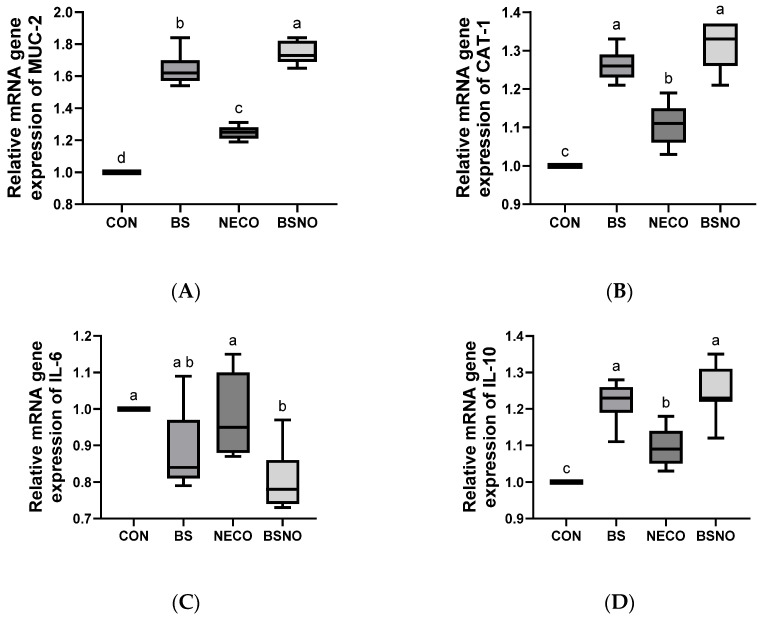
Effect of *B. subtilis*, nano-encapsulated cumin oil, and their mixture supplementation on intestinal gene expression (mucin-2 (MUC-2, (**A**))), cationic amino acid transporter-1 (CAT-1, (**B**)), interleukin-6 (IL-6, (**C**)) and interleukin-10 (IL-10, (**D**)) of growing rabbits at 77 d. CON: a basal diet without a feed additive (control diet), BS: control diet plus *B. subtilis* (500 mg/kg), NECO: control diet plus nano-encapsulated cumin oil (200 mg/kg), BSNO: control diet plus *B. subtilis* and nano-encapsulated cumin oil. Values with different superscript letters are statistically different.

Experimental supplements showed a positive effect on intestinal gene expression in rabbits exposed to heat stress, as shown in Figure 7A–D. Gene expression of MUC-2, CAT-1, and IL-10 increased in the BS, NECO, and BSNO groups compared to the control group (*p* < 0.05), while MUC2 gene expression was highest in the BSNO group, also CAT-1 and IL-10 gene expression was highest in the BS and BSNO groups (*p* < 0.05). Additionally, IL-6 gene expression decreased in the BSNO groups compared to the control, BS, and NECO groups (*p* < 0.05).

## 4. Discussion

Recently, there has been increased interest in nutritional manipulation as a novel strategy to improve the health, performance, and efficiency of rabbits against the effects of heat stress [3]. Feed additives such as a mixture of *B. subtilis* and nano-encapsulated cumin oil could be an effective additive to alleviate the harmful effects of heat stress in rabbits. In particular, the results of the current study showed that rabbits were exposed to heat stress through increased THI values, accompanied by increased respiratory and pulse rates. Notably, both RR and PR increased significantly during periods of high THI, reflecting the rabbits’ thermal sensitivity to high ambient temperature and humidity, which puts them at risk for heat stress. It is interesting to note that the addition of *B. subtilis* and nano-encapsulated cumin oil mixture alleviated the harmful effects of heat stress in rabbits, which may be due to their multiple biological properties, including interaction with the gut environment, immunomodulatory activities, antioxidant system, and antimicrobial activity.

The results of the current study showed a significant improvement in growth performance in rabbits receiving a mixture of *B. subtilis* and nano-encapsulated cumin oil during heat stress via increased BWG and decreased FCR. Consistent with our study, growth performance was enhanced in rabbits fed a diet including probiotics [32]. Likewise, feeding rabbits a diet containing essential oils significantly improved body weight and feed conversion ratio and decreased the mortality rate [19,33,34]. In a similar study, Alimohamadi et al. [35] found that adding cumin powder and probiotics to the diet significantly improved broilers’ growth performance. The improved growth of heat-stressed rabbits fed essential oils can be attributed to the role of bioactive compounds (such as cuminaldehyde, γ-terpinene, and β-pinene) in nano-encapsulated cumin oil [17]. This can be attributed to several mechanisms, including the stimulation of digestive enzyme secretion and the production of bile acids, which enhance digestion and nutrient absorption [17,18,36]. Furthermore, they have antioxidant and anti-inflammatory properties [33,37], promote the growth of beneficial bacteria in the gut [18], modify intestinal morphology [19], and enhance the activity of digestive enzymes [8,23], thereby increasing feed utilization and improving growth. Additionally, several previous reports have shown that adding *B. subtilis* to the diets of animals alters the balance of intestinal microbes, modifies and stimulates immune function [16,38], competes for chemicals and adhesion sites on epithelial cells [12,39], as well as produces compounds that inhibit the growth of pathogenic microbes [17], thus enhancing rabbit health and performance. However, some reports purport that essential oils did not affect growth performance in broiler chickens [40,41]. This discrepancy between study results may be attributed to the chemical composition of the essential oils used, the extraction method, the amounts added, the conditions of addition, the animal type and age, and the experimental conditions. From the above, it is clear that the synergistic effect of nano-encapsulated cumin oil with *B. subtilis* supplementation in enhancing intestinal integrity, oxidative stability, immune response, and intestinal microbial modification is evident, which increases the availability of nutrients and the activity of digestive enzymes. Therefore, the addition of the mixture can play an effective role in enhancing antioxidant defenses, gut health, and nutrient efficiency, supporting the animals’ ability to adapt to heat stress, leading to improved growth performance and significantly reduced mortality rates in growing rabbits exposed to heat stress.

Despite the significant improvement in growth performance, this study found that carcass characteristics were not affected except for carcass weight, which increased in rabbits fed a diet containing a mixture of nano-encapsulated cumin oil and *B. subtilis*. Yilmaz and Gul [35] found an increase in carcass weight in stressed broilers fed a diet including cumin essential oil, which is in agreement with our results. Additionally, Fathi et al. [42] and Mohamed et al. [43] indicated that carcass characteristics of rabbits receiving dietary probiotics, whether *B. subtilis* or a mixture of *Lactobacillus acidophilus* and *Bifidobacterium bifidum*, improved; however, Tufarelli et al. [44] did not find an effect of probiotic supplements on carcass characteristics. Variations in essential oil type, concentration, and experimental conditions may explain the inconsistent findings. Some reports indicate that the positive role of probiotic supplements in enhancing carcass weight may be due to their effect on the weight and function of the digestive system [33], as well as the cecum microbial fermentation pattern in rabbits [13], which increases nutrient availability, as well as adding essential oil enhancing activity of digestive enzymes and digestion of nutrients [17,37], thus supporting carcass weight gain.

Data from the current study indicated a decrease in the digestibility of nutrients in rabbits with increasing ambient temperature, which is confirmed by many previous reports [3]. Moreover, the results of the current study showed that adding a nano-encapsulated cumin oil with *B. subtilis* mixture increased the digestibility of fiber and protein and secretion of trypsin enzymes. Consistent with these findings, Elbaz et al. [8] and Phuoc and Jamikorn [16] reported significantly increased digestibility of crude fiber and protein in chickens fed a diet including essential oils or probiotics. The positive effect of the mixture may be attributed to the role of the probiotic in promoting gut health by modifying the microbial content, reducing inflammation, and enhancing oxidative stability and intestinal morphology [33,45]. In agreement with our findings, some reports indicate that essential oils, through their bioactive compounds, stimulate the secretion of digestive enzymes, which enhances nutrient digestibility [46,47]. These compounds stimulate the secretion of digestive hormones (such as cholecystokinin (CCK) and secretin), which activate pancreatic exocrine function, enhance enzyme synthesis, and bile acids, improving lipid and carbohydrate digestion [19,48]. Improving gut health (improving gut morphology, reducing pathogenic bacteria, and oxidative stress) and enhancing nutrient signaling also stimulates the pancreas to increase digestive enzyme production [46,49], creating a more favorable environment for nutrient digestion and absorption. Additionally, its antioxidant and anti-inflammatory properties protect intestinal and pancreatic tissue, contributing to overall gut health and stimulating pancreatic activity, which together increase the synthesis and secretion of digestive enzymes [48,49]. This demonstrates the effective effect of adding a combination of nano-encapsulated cumin oil and *B. subtilis* to enhance nutrient digestion, thus improving feed utilization and performance in heat-stressed rabbits.

The results of the current study indicate that the addition of nano-encapsulated cumin oil and *B. subtilis* has a significant effect on protein, carbohydrate, and lipid metabolism in heat-stressed rabbits. Nano-encapsulated cumin oil with *B. subtilis* mixture reduced lipid metabolism, while enhancing protein metabolism, as demonstrated by increased serum total protein and HDL levels and decreased triglycerides and cholesterol levels. A similar study demonstrated that the addition of essential oils had a significant effect on decreasing levels of LDL, triglycerides, and cholesterol [10,34,35]. Moreover, Tufarelli et al. [50] reported that the addition of probiotics played an effective role in modifying fat metabolism in poultry during heat stress by reducing levels of triglycerides and cholesterol. The lipid-lowering effects of the blend of probiotic and essential oil can be attributed to the blend’s ability to improve lipid metabolism and digestion [37] through several mechanisms, like negatively impacting the activity of enzymes involved in cholesterol synthesis and metabolism (cholesterol 7-alpha-hydroxylase (CYP7A1) and 3-hydroxy-3-methylglutaryl-CoA reductase (HMGCR) [46,47]. Additionally, heat stress leads to an increase in free radicals and a decrease in antioxidant defenses, causing oxidative damage that harms the liver [2]. To confirm the impact of heat stress and experimental supplements on liver function and health, levels of liver enzymes such as AST and ALT were measured. Our results showed decreased AST levels in rabbits that received a nano-encapsulated cumin oil and *B. subtilis* mixture. On the other hand, adding experimental supplements in the current study mitigated these harmful effects on the liver by reducing plasma AST levels [50]. In the same context, Moustafa et al. [51] found that adding essential oils reduced the AST level in chickens subjected to heat stress. The lower AST levels in poultry receiving essential oils may be attributed to the beneficial effects of bioactive compounds, including preventing protein degradation, increasing the production of antioxidant enzymes, and improving the functions and integrity of tissues and organs [16,19]. Moreover, the bioactive compounds in essential oils possess strong antioxidant and anti-inflammatory properties that exert hepatoprotective effects by stabilizing cell membranes, reducing oxidative damage in the liver, and thereby preventing the leakage of intracellular enzymes such as AST into the bloodstream [52]. These findings indicate that the addition of the mixture may have a positive effect on hepatic function and health.

Additionally, the many negative effects of heat stress on glandular performance reduce thyroid activity and consequently reduce the production of its hormones, namely triiodothyronine (T3) and thyroxine (T4), which leads to a decrease in energy production and metabolic rate [53], which contributes to weakening immune function, deterioration of general health, and decreased growth. This is part of a physiological adaptation: reducing basal metabolic rate to decrease internal heat production [3]. The results are consistent with previous reports, where T3 and T4 levels were decreased in rabbits exposed to heat stress and fed a diet without additives [22,23,24,25,26,27,28,29,30,31,32,33,34,35,36,37,38,39,40,41,42,43,44,45,46,47,48,49,50,51,52,53,54], as in the present study. However, T3 and T4 levels increased in rabbits that received nano-encapsulated cumin oil and *B. subtilis* mixture. Several previous reports support the beneficial effect of probiotic supplements and essential oils in maintaining thyroid function by increasing the production of thyroid hormones [22,54], which enhances nutrient metabolism and improves growth performance. Combining probiotics with essential oils may have synergistic effects in supporting endocrine function, including the thyroid. Probiotics may help maintain or restore thyroid hormone levels by mitigating these effects by reducing stress (e.g., corticosterone) [29,38], improving gut health, and enhancing nutrient absorption. Besides that, essential oils reduce oxidative and inflammatory damage, protect cellular integrity (including thyroid tissue), and support metabolic function [19,33,35]. Some studies combining probiotics with essential oils show improvements in metabolic biomarkers, growth performance, and overall health in chickens [5,55].

One of the most significant harms of heat stress is the decrease in antioxidant defenses and the increase in free radicals, which cause oxidative damage that affects many organs, including the liver and intestines, as well as lipid peroxidation [33,36]. Antioxidant enzymes are the primary line of defense to reduce the damage caused by oxidative stress. The results of the current study showed that adding a nano-encapsulated cumin oil and *B. subtilis* mixture increased the activity of antioxidant enzymes, such as SOD. In agreement with our results, several studies have reported that the addition of probiotics or essential oils enhanced the oxidative status by increasing antioxidant enzymes, including SOD, GPx, and TAC [22,56]. In addition, previous studies have shown that several experimental supplements, including essential oils and probiotics, had a beneficial effect in maintaining cell integrity during heat stress in rabbits or chickens, as indicated by decreased MDA levels [44,56,57], which is consistent with our results. Cumin essential oil is rich in bioactive compounds with powerful free radical scavenging properties [17]. These compounds directly neutralize reactive oxygen species (ROS), as well as stimulate the nuclear factor erythroid 2-related factor 2 (Nrf2) signaling pathway, which regulates gene expression encoding antioxidant enzymes [58,59,60], improving oxidative stability and the rabbit’s ability to tolerate heat stress conditions. Meanwhile, *B. subtilis* improves gut health and nutrient absorption, enhancing rabbits’ oxidant stress by suppressing harmful microbes, thereby reducing reactive oxygen species. Furthermore, *B. subtilis* can produce several compounds (such as exopolysaccharides, short-chain fatty acids, and certain enzymes) [12,29] that stimulate antioxidant activity in rabbits. The addition of nano-encapsulated cumin oil with *B. subtilis* mixture may have a protective role against heat stress by supporting oxidative stability in growing rabbits.

In this study, we investigated whether adding nano-encapsulated cumin oil with *B. subtilis* mixture supplements could enhance immune responses in heat-stressed rabbits. Therefore, immunoglobulin levels were assessed as indicators of the effectiveness of these supplements on immune responses, especially since immunoglobulins are present in the mucosal regions of the respiratory and gastrointestinal tracts, which limits the colonization of pathogens. The current study showed that immunoglobulin levels increased significantly, particularly IgG and IgA, when supplemented with nano-encapsulated cumin oil and *B. subtilis* mixture to rabbit diets under heat stress. Similar results were found by Abdel-Moneim et al. [57] and Humam et al. [60], with increased levels of IgA and IgG in the blood of rabbits receiving probiotics. Similarly, Elbaz et al. [22,61] found that chickens receiving a mixture of essential oils and probiotics increased IgG levels. Cumin essential oil is characterized by its potent bioactive compounds, which have immunomodulatory properties. They help neutralize ROS and reduce oxidative stress [58], thus preserving immune cell function. They also stimulate cytokine secretion and the activity of macrophages and lymphocytes, leading to improved humoral and cellular immune responses [12,14]. Additionally, *B. subtilis* acts as an effective probiotic immunomodulatory by improving intestinal microbial balance and stimulating the gut-associated lymphoid tissue [62,63], the primary site of immune regulation in poultry. The addition of *B. subtilis* also enhances B-lymphocyte proliferation and antibody synthesis, leading to increased levels of immunoglobulins (IgA, IgG, and IgM), as well as strengthening the intestinal mucosal barrier by increasing the production of secretory immunoglobulin (sIgA), which represents the first line of defense against intestinal pathogens [62]. From the above, the observed improvement in immune response may be attributed to the antimicrobial and anti-inflammatory properties of nano-encapsulated cumin oil and *B. subtilis* mixture [14,39], which helps in providing nutrients and thus promoting the proliferation of lymphocytes in primary immune organs and enhancing intestinal integrity [64], thus stimulating the production of immunoglobulins and the immune response.

Heat stress damages the intestine by changing the microbial community (increasing harmful bacteria), immune response, and gene expression of the intestinal barrier [3], thus damaging the intestinal barrier, epithelial cells, and microstructure, which negatively affects the function of the digestive system in digesting and absorbing nutrients [65], leading to poorer rabbit performance. This is consistent with our results, where the number of harmful microbes (*C. perfringens* and *E. coli*) increased and the expression of intestinal integrity-related genes declined. Creating a healthy digestive environment is essential for rabbits to maintain their health and productive performance [3,9], as well as to resist intestinal disturbances and environmental changes through experimental supplements. Furthermore, several reports have indicated that experimental additives had a positive effect in maintaining intestinal health by modifying microbial content and gene expression [31,66]. Consistent with this, the current study showed that adding a nano-encapsulated cumin oil and *B. subtilis* mixture led to an increase in *Lactobacillus* counts and gene expression of MUC-2, CAT-1, and IL-10, while decreasing *E. coli* and *C. perfringens* counts and gene expression of IL-6. The impacts of essential oils and probiotics on pathogenic microorganisms can be attributed to several distinct mechanisms, including altering their adhesion to the epithelium, altering the pH of the intestinal lumen, reducing the production of toxic compounds, preventing biofilm formation [37,44], and inhibiting the growth of pathogenic microbes [54] by interacting with microbial cell membranes, causing cell disruption. The antimicrobial activity of essential oils can also be attributed to their bioactive compounds, which disrupt nucleic acid synthesis and ATPase, altering cell membrane permeability [37] and thus exhibiting antibacterial effects against pathogens.

Identifying changes in gene expression patterns of genes that play a vital role in rabbit health and performance is a critical measure for assessing the impact of heat stress and experimental nutritional supplements on rabbit performance [8,67]. High ambient temperature has a gene-modifying effect, negatively impacting nutrient absorption and stimulating various defense activities to protect various tissue cells [66], including immune-related genes (cytokines). The current study demonstrated the impact of heat stress on the gene expression of several genes, decreased expression of MUC-2, CAT-1, and IL-10 gene, while increased expression of IL-6, indicating induced inflammation, consistent with many previous studies. Additionally, many previous reports have indicated the positive role of feed additives, including essential oils and probiotics, in modulating the expression of genes related to growth, inflammation, and immune response, as well as genes regulating digestion, absorption, and intestinal health and metabolism in chickens and rabbits [22,54]. Consistent with previous reports, the nano-encapsulated cumin oil and *B. subtilis* mixture had a significant effect on gene expression, increasing expression of IL-10, CAT-1, and MUC-2 genes, while decreasing expression of IL-6. Similarly, previous reports found that adding essential oils had a modulatory effect on the MUC-2 and IL-10 genes [68]. Moreover, Yosi and Metzler-Zebeli [68], and Abdel-Raheem et al. [69] found increased MUC-2 and IL-10 gene expression in chickens fed a probiotic diet. The influence of essential oil and probiotic supplementation on gene expression in rabbits may be attributed to their antimicrobial activity, which positively alters the intestinal microbiome, promotes intestinal growth and immune function, maintains the integrity of the intestinal barrier, and reduces inflammation, all of which contribute to enhanced intestinal health and growth. Additionally, mucus is the first line of immune defense within the gastrointestinal tract, and enhancing its secretion has a beneficial effect in preventing the invasion of pathogenic microbes and their toxins [70]. Hence, the combination of nano-encapsulated cumin oil with *B. subtilis* could provide synergistic functions to enhance intestinal integrity by counteracting the inflammatory response and enhancing mucus secretion, providing a promising avenue for enhanced health benefits in rabbits.

## 5. Conclusions

This study demonstrates that adding a mixture of nano-encapsulated cumin oil and *B. subtilis* has a positive effect in reducing the impacts of heat stress in growing rabbits by enhancing oxidative stability and immune response, as well as improving lipid profile and thyroid gland performance. Meanwhile, the mixture enhanced gut health, including reducing inflammation and pathogenic microbes, and up-regulated the expression of the mucin-2 (MUC-2) gene. This mixture also contributed to increased nutrient digestibility and up-regulation of the cationic amino acid transporter-1 (CAT-1) gene expression, which enhanced feed utilization efficiency and carcass weight. Therefore, adding a mixture of nano-encapsulated cumin oil and *B. subtilis* to the diet of growing rabbits could be beneficial for improving growth performance as an effective anti-stress supplement.

## Figures and Tables

**Table 1:** Chemical Composition of the CEO.

Ingredients	%
Cuminaldehyde	38.6
p-cymene	19.3
β-pinene	8.2
α-terpinen-7-al	11.5
γ-terpinene	7.8
p-cymen-7-ol	5.7
thymol	3.2
other components	5.7

**Table 2:** Composition of basal diet for rabbits and chemical analysis.

Ingredients	(%)
Yellow corn	15.8
Clover hay	32.0
Soybean meal (44%)	14.6
Sunflower meal	5.00
Wheat bran	12.0
Barley	13.0
Di- Calcium phosphate	2.10
Limestone	1.50
NaCl	0.50
Premix (Vit-Min) *	0.50
Molasses	3.00
Nutrient level	
Dry matter	89.3
Energy (kcal/kg)	2468
Crude protein (%)	16.7
Ether extract	3.21
Crude fiber (%)	12.4
Calcium (%)	1.36
Phosphor (%)	0.68

* Each 1 kg of vitamin-mineral premix contained: 5460 mg of phylloquinone, 13,130 mg of DL-3-tocopheryl acetate, 5640 mg of thiamine, 14,560 mg of riboflavin, 7350 mg of pyridoxine, 27,300 mg of Ca-D-pantothenate, 3640 mg of folic acid, 10,920 mg of niacin, 29.12 mg of cobalamin, 12,000 mg of manganese, 3000 mg of selenium, 2370 mg of D-biotin, 900 mg of zinc, 160 mg of copper, 12,500 mg of iodine, and 40,000 mg of ferrous.

**Table 3:** Effect of *B. subtilis*, nano-encapsulated cumin oil, and their mixture supplementation on growth performance of growing rabbits.

Parameter	CON	BS	NECO	BSNO	SEM	*p* Value
IBW, g	781	780	783	781	12.35	0.942
Period 35–56 d						
BWG, g/d	20.8 ^b^	21.9 ^a,b^	21.5 ^a,b^	23.1 ^a^	1.22	0.016
DFI, g/d	32.3	32.6	32.4	32.9	1.81	0.287
FCR, g:g	1.97 ^a^	1.91 ^a^	1.93 ^a^	1.86 ^b^	0.08	0.030
Period 57–77 d						
BWG, g/d	25.2 ^c^	26.8 ^b^	26.3 ^b^	27.6 ^a^	2.14	0.001
DFI, g/d	58.4	57.7	59.2	58.0	3.09	0.431
FCR, g:g	2.31 ^a^	2.15 ^b^	2.26 ^a,b^	2.10 ^b^	0.34	0.002
Period 35–77 d						
BWG, g/d	23.1 ^c^	24.4 ^b^	23.9 ^b,c^	25.3 ^a^	1.18	<0.001
DFI, g/d	90.7	90.3	91.4	90.9	2.64	0.627
FCR, g:g	3.93 ^a^	3.70 ^c^	3.83 ^b^	3.58 ^d^	0.13	<0.001

^a–d^ Means with different superscripts in the same row indicate significant differences (*p* < 0.05), CON: rabbits fed a basal diet without a feed additive (control diet), BS: control diet plus *B. subtilis* (500 mg/kg), NECO: control diet plus nano-encapsulated cumin oil (200 mg/kg), BSNO: control diet plus *B. subtilis* and nano-encapsulated cumin oil, BWG: body weight gain, IBW: initial body weight, DFI: feed intake, FCR: feed conversion ratio. SEM: standard error of the mean.

**Table 4:** Effect of *B. subtilis*, nano-encapsulated cumin oil, and their mixture supplementation on carcass characteristic of growing rabbits at 77 d.

Parameter	CON	BS	NECO	BSNO	SEM	*p* Value
LBW, g	1903 ^d^	2140 ^b^	2038 ^c^	2197 ^a^	12.9	<0.001
Carcass, %	58.4 ^c^	61.2 ^b^	60.7 ^b^	63.1 ^a^	6.35	0.001
Heart, %	0.29	0.30	0.29	0.28	0.05	0.105
Kidney, %	0.70	0.68	0.71	0.70	0.09	0.097
Lungs, %	0.69	0.70	0.68	0.71	0.13	0.211
Liver, %	3.15	3.13	3.23	3.19	1.04	0.094
Giblets, %	4.21	4.31	4.28	4.26	0.62	0.307
TEP, %	59.7	60.6	60.1	60.8	3.07	0.128

^a–d^ Means with different superscripts in the same row indicate significant differences (*p* < 0.05), CON: a basal diet without a feed additive (control diet), BS: control diet plus *B. subtilis* (500 mg/kg), NECO: control diet plus nano-encapsulated cumin oil (200 mg/kg), BSNO: control diet plus *B. subtilis* and nano-encapsulated cumin oil, LBW: live body weight, TEP: Total edible parts. SEM: standard error of the mean.

**Table 5:** Effect of *B. subtilis*, nano-encapsulated cumin oil, and their mixture supplementation on digestibility coefficients (%) of growing rabbits at 77 d.

Parameter	CON	BS	NECO	BSNO	SEM	*p* Value
Dry matter	63.2 ^b^	64.8 ^a^	63.8 ^a,b^	65.4 ^a^	2.041	0.016
Crude fiber	42.8 ^c^	45.1 ^a,b^	44.3 ^b^	45.8 ^a^	0.845	0.009
Crude protein	68.7 ^d^	71.3 ^b^	70.2 ^c^	72.5 ^a^	1.106	0.024
Ether extract	81.6	81.8	82.4	82.7	3.577	0.072
NFE	52.3	51.8	52.7	52.5	1.635	0.161

^a–d^ Means with different superscripts in the same row indicate significant differences (*p* < 0.05), CON: a basal diet without a feed additive (control), BS: control diet plus *B. subtilis* (500 mg/kg), NECO: control diet plus nano-encapsulated cumin oil (200 mg/kg), BSNO: control diet plus *B. subtilis* and nano-encapsulated cumin oil, NFE: nitrogen-free extract. SEM: standard error of the mean.

**Table 6:** Effect of *B. subtilis*, nano-encapsulated cumin oil, and their mixture supplementation on blood biochemical profile (%) of growing rabbits at 77 d.

Parameter	CON	BS	NECO	BSNO	SEM	*p* Value
Total protein, g/dL	6.74 ^b^	6.83 ^a,b^	6.94 ^a^	6.92 ^a^	0.204	0.041
Albumin, g/dL	3.18	3.21	3.19	3.23	0.133	0.110
Triglycerides, mg/dL	64.3 ^a^	61.7 ^b,c^	62.6 ^b^	60.8 ^c^	2.091	0.006
Cholesterol, mg/dL	72.8 ^a^	68.4 ^c^	71.3 ^b^	67.9 ^c^	3.770	<0.001
LDL, mg/dL	21.3	20.8	20.2	19.8	0.975	0.072
HDL, mg/dL	24.1 ^c^	25.6 ^b^	25.3 ^b^	26.6 ^a^	0.624	0.001
ALT, U/L	14.9	15.3	15.4	15.1	0.520	0.327
AST, U/L	53.3 ^a^	51.2 ^b^	51.7 ^b^	49.6 ^c^	1.208	0.001

^a–c^ Means with different superscripts in the same row indicate significant differences (*p* < 0.05), CON: a basal diet without a feed additive (control diet), BS: control diet plus *B. subtilis* (500 mg/kg), NECO: control diet plus nano-encapsulated cumin oil (200 mg/kg), BSNO: control diet plus *B. subtilis* and nano-encapsulated cumin oil, HDL: high-density lipoprotein, LDL: low-density lipoprotein. SEM: standard error of the mean.

**Table 7:** Effect of *B. subtilis*, nano-encapsulated cumin oil, and their mixture supplementation on cecum microbial enumeration (Log10 CFU g^−1^) of growing rabbits at 77 d.

Parameter	CON	BS	NECO	BSNO	SEM	*p* Value
*Lactobacillus*	5.44 ^c^	6.61 ^a,b^	6.04 ^b^	7.03 ^a^	0.974	<0.001
*E. coli*	4.78 ^a^	3.71 ^c^	4.16 ^b^	2.94 ^d^	1.022	<0.001
*C. perfringens*	3.89 ^a^	2.78 ^b^	3.67 ^a^	2.45 ^b^	0.540	0.001

^a–d^ Means with different superscripts in the same row indicate significant differences (*p* < 0.05), CON: a basal diet without a feed additive (control diet), BS: control diet plus *B. subtilis* (500 mg/kg), NECO: control diet plus nano-encapsulated cumin oil (200 mg/kg), BSNO: control diet plus *B. subtilis* and nano-encapsulated cumin oil, *C. perfringens: Clostridium perfringens*, and *E. coli: Escherichia coli*. SEM: standard error of the mean.

## Data Availability

The original contributions presented in this study are included in the article. Further inquiries can be directed to the corresponding authors.

## Abbreviations

The following abbreviations are used in this manuscript:

ALT	Alanine aminotransferase
AST	Aspartate aminotransferase
BS	Growing rabbits received a basal diet with *Bacillus subtilis*
BSNO	Growing rabbits received a basal diet with *Bacillus subtilis* and nano-encapsulated cumin oil
BWG	Body weight gain
*C. perfringens*	*Clostridium perfringens*
CAT-1	Cationic amino acid transporter-1
CON	Growing rabbits received a basal diet
*E. coli*	*Escherichia coli*
FCR	Feed conversion ratio
FI	Feed intake
GPx	Glutathione peroxidase
HDL	high-density lipoprotein
IL-10	Interleukin 10
IL-6	Interleukin 6
LDL	low-density lipoprotein
MDA	Malondialdehyde
MUC-2	Mucin 2
NECO	Growing rabbits received a basal diet with nano-encapsulated cumin oil
PR	Pulse rate
RR	Respiratory rate
SOD	Superoxide dismutase
T3	Triiodothyronine
T4	Thyroxine
THI	Temperature-humidity index
